# Editorial: Large language models in work and business

**DOI:** 10.3389/frai.2024.1516832

**Published:** 2024-11-29

**Authors:** Şadi Evren Şeker

**Affiliations:** Computer Science and Engineering, Istanbul University, Istanbul, Türkiye

**Keywords:** LLM, AI in business, XAI, knowledge graph, data driven decision making

In today's rapidly evolving business landscape, Artificial Intelligence (AI), and specifically Large Language Models (LLMs), are redefining how organizations operate, make decisions, and engage with customers. AI-driven technologies have become indispensable, providing businesses with powerful tools to streamline operations, derive actionable insights from vast data, and foster more meaningful customer interactions. For business leaders, scholars, and practitioners alike, understanding the transformative potential of AI isn't just advantageous—it's essential to staying competitive in an increasingly data-driven world.

This editorial delves into recent scholarly advancements in LLM applications within business contexts, analyzing studies that explore AI's potential across various domains, from decision support to creative industries. By introducing a structured framework, this editorial highlights key insights and contributions from recent studies, assessing their value to academia and industry. The following comparative analysis sheds light on how these innovations shape our understanding of AI's role in business while pointing to future research directions.

Puyt and Madsen's study stands out as a foundational exploration of LLM accuracy, assessing ChatGPT-4's ability to recount the history of the SWOT analysis—a vital business strategy tool. Their findings reveal that, while ChatGPT-4 effectively conveys general concepts, it struggles with detailed historical information, often producing inaccuracies or “hallucinations.” This gap underscores the need for LLMs to be trained with verified academic data, particularly for strategic business applications that demand precision. This study not only contributes to the literature by proposing methods to evaluate AI accuracy in historical contexts but also highlights the importance of rigorous information vetting in industry settings where reliability is crucial.

In contrast, Raikov et al. explore a hybrid intelligence model that combines LLM capabilities with explainable AI (XAI) principles to enhance human-machine collaboration. Their approach emphasizes cognitive semantics, improving transparency and decision-making efficiency. The hybrid model's real-time adaptability addresses the needs of complex, regulated industries such as finance and healthcare, where trust in AI decisions is paramount. Academically, this study provides a valuable addition to XAI literature by demonstrating how LLMs can bridge the gap between AI autonomy and human oversight, making it a model for future human-AI interactions in complex business environments.

Another significant study by Mariotti et al. examines the integration of LLMs with enterprise knowledge graphs to enhance data-driven decision-making. By enabling organizations to leverage knowledge graphs for more accurate and scalable data retrieval, this research provides a robust framework for businesses seeking efficient knowledge management systems. The academic contribution here lies in advancing the dialogue between LLMs and knowledge graphs, emphasizing ethical data handling and quality standards essential for industry applications. For enterprises, the study offers practical solutions to achieve streamlined data management, balancing automation with privacy and security.

Ryjov et al. take a different approach, investigating LLMs' role in creative industries, specifically within fashion design. They introduce a hybrid intelligence model that supports creative processes, allowing AI to complement rather than replace human ingenuity. While LLMs in this field demonstrate potential in automating repetitive design tasks and enhancing customer personalization, the study reveals limitations in AI's ability to handle spatial and stylistic nuances. This study's academic contribution lies in promoting human-AI co-creation, inspiring further research into AI applications across diverse creative sectors, including media and marketing.

Collectively, these studies not only illuminate LLMs' transformative potential in business but also highlight critical ethical and operational considerations. Ensuring accuracy, transparency, and data privacy are vital to responsibly integrating AI into business workflows. Future research should focus on enhancing LLM accuracy, refining hybrid intelligence models, and exploring creative AI applications, all while maintaining ethical standards. As LLMs evolve, interdisciplinary collaborations will be essential to harness their full potential, making AI an ethical, effective, and innovative force in the business world.

